# Body Compression Corrective Garment and Eating Behavioural Change for Weight Reduction: The Mutsu City Randomised Controlled Trial

**DOI:** 10.3390/healthcare11070942

**Published:** 2023-03-24

**Authors:** Akira Kanda, Yoshikuni Sugimura, Hideki Ohishi, Satoru Tatebayashi, Kaori Sawada, Kyi Mar Wai, Kei Nishiguchi, Asano Tanabu, Songee Jung, Koichi Murashita, Shigeyuki Nakaji, Kazushige Ihara

**Affiliations:** 1 Department of Health and Beauty Science, Graduate School of Medicine, Hirosaki University, Aomori 036-8562, Japan; a_kanda@auhw.ac.jp (A.K.); h.ooishi@atsugi.co.jp (H.O.); nakaji@hirosaki-u.ac.jp (S.N.); 2 Department of Nutrition, Faculty of Health Sciences, Graduate School of Health Sciences, Aomori University of Health and Welfare, Aomori 030-8505, Japan; 3 Department of Social Medicine, Graduate School of Medicine, Hirosaki University, Aomori 036-8562, Japan; y.sugimura@hirosaki-u.ac.jp (Y.S.); iwane@hirosaki-u.ac.jp (K.S.); kyimar@hirosaki-u.ac.jp (K.M.W.); h17gm141@hirosaki-u.ac.jp (A.T.); 4 Department of Innovation Center for Health Promotion, Graduate School of Medicine, Hirosaki University, Aomori 036-8562, Japan; 5 Atsugi Corporation, Kanagawa 243-0493, Japan; s.tatebayashi@atsugi.co.jp; 6 Atsugi Tohoku Mutsu Office, Aomori 035-0061, Japan; kei.nishiguchi.3j@hirose-gl.com; 7 Department of Digital Nutrition and Health Sciences, Graduate School of Medicine, Hirosaki University, Aomori 036-8562, Japan; jonsoni@hirosaki-u.ac.jp; 8 COI Research Initiatives Organization, Graduate School of Medicine, Hirosaki University, Aomori 036-8562, Japan; murasita@hirosaki-u.ac.jp

**Keywords:** body compression corrective garment, obesity, behaviour change programme, self-esteem

## Abstract

Affordable and accessible behaviour-based interventions that do not overwhelm or demoralise overweight/obese individuals are needed. Combining clothing with behaviour change techniques might be an option. This is because clothing is a social norm, and clothing and motivation for weight loss are associated with the common desire to look better. Therefore, we conducted a single-blind randomised controlled trial to examine the effect of an intervention that combined behaviour change techniques, including simplified goal setting and self-monitoring, with a body compression corrective garment (BCCG), which exerts continuous but minimal tactile pressure on the hips and abdomen. We enrolled healthy community-dwelling adults with a body mass index ≥ 25 kg/m^2^ and assigned 35 and 34 participants to the intervention and control groups, respectively. The reduction in body weight was 1.3 kg more in the intervention group than in the control group after the 12-week intervention period (*p* < 0.05, repeated-measures mixed model). In addition, eating behaviour and body appreciation showed significant improvement in the intervention group compared with the control group. Our newly developed intervention improved eating behaviour and body appreciation and reduced the body weight of overweight/obese participants. Wearing a BCCG seems to facilitate behavioural changes and lead to weight loss.

## 1. Introduction

Obesity is a well-known cause of cardiometabolic diseases and their mortality. The global increase in the prevalence of obesity is a major public health issue [1]. The prevalence of obesity (body mass index [BMI] ≥ 25 kg/m^2^) in Japan has risen at an alarming rate over the past 30 years, reaching 33.0% in men and 22.3% in women, despite public awareness of the associated risk of chronic diseases [2]. Awareness includes lifestyle changes, such as reducing excess dietary calorie intake and physical energy expenditure. Decreasing the prevalence of obesity is a matter of concern in the general population, and finding an effective approach for individuals is essential for weight reduction.

Various nonpharmacological interventions to reduce weight through lifestyle changes have been developed in community and primary care settings. Behaviour-based interventions, including behaviour change techniques such as motivation, goal setting, and self-monitoring, constitute the main part of the interventions. Among the different behaviour change techniques, multi-component techniques have been reported to be more effective than single techniques [3,4]. However, multi-component behaviour techniques are often costly and time-constrained and require various resources to encourage behaviour change and achieve weight loss. They require professionals with high counselling skills and knowledge of behaviour change who are not always available in the community and primary care settings [5,6]. The barriers for overweight/obese people to engage in behaviour change programmes include the inconvenience caused by meetings, lack of time or transportation, feelings of embarrassment, and financial costs [7]. Behaviour-based interventions that are accessible and rely less on counsellors or coaches are needed for both providers and obese/overweight individuals.

Motivation is key for participants to start and maintain behaviour-change programmes [8]. Looking appealing and feeling assured are the most important factors that motivate participants. Looking better and being healthier are positive reasons for obese/overweight individuals to participate in a behaviour change programme [8]. In addition, clothing is a possible intervention, as wearing clothes that an individual prefers can help generate a positive feeling about appearance and consequently improve autonomy, self-esteem, and self-efficacy. Clothes play essential roles in conserving body heat and adjusting the absorption of light [9]. They are also functionally utilised for recovery from injury and enhancement of athletic performance. For example, wearing a garment that facilitates body compression was found to lower the mean body temperature during recovery after cycle ergometer trials [10] and aid in preventing knee injuries from drop vertical jumping [11]. Body compression garments are widely used in medical, athletic, and body-shaping applications [12], while heavy pressure, such as that exerted by a tightly laced corset, has an unhealthy effect. However, we found no previous study on body compression garments used for intervention in overweight/obesity.

We introduced a body compression corrective garment (BCCG), developed to integrate positive appearance, good health, and cognitive-behavioural effects into conventional weight reduction intervention programmes for a lifestyle change. We expected this BCCG to function as a motivational tool. This new intervention, including the daily wearing of the BCCG, may prompt participants to consciously feel their body shape and facilitate sustainable efforts to change their lifestyle. Therefore, the prospect of a more appealing physical appearance may be a powerful motivator for behavioural change [13]. Therefore, the aim of this study was to determine the effects of a trial in which BCCG was incorporated into behaviour change techniques over 12 weeks of body weight reduction.

## 2. Materials and Methods

### 2.1. Design

In collaboration with the Hirosaki University Graduate School of Medicine and Mutsu Municipal Government, a single-blind parallel randomised controlled trial (RCT) with an allocation ratio of 1:1 was conducted in Mutsu City, located in the northeast of Aomori Prefecture, Japan. The Department of Health and Beauty Science at Hirosaki University and the Health Promotion Office at Mutsu Municipal Government were responsible for the recruitment and data collection from participants at baseline and follow-up. The RCT was named ‘Mutsu Shi Cute Kyoshitsu’ (Mutsu City Cute Course). The semantic meaning of the term ‘cute’ in Japanese has shifted, and the term does not have the same meaning as that in English. The word ‘cute’ is a homonym for ‘kyutto’ (kyúto) in Japanese, which means ‘pressed out extra weight’ or ‘racked some ideas out from the brain.’ The name of the RCT was derived from the study’s aim.

This study was approved by the Ethics Committee of the Graduate School of Medicine, Hirosaki University (registration number 2019-090) and the University Hospital Medical Information Network Clinical Trials Registry (registration number UMIN000041327). The first registration date was 5 August 2020. All research activities were performed in accordance with relevant guidelines/regulations.

### 2.2. Recruitment and Participants

The recruitment was conducted from March to August 2020. Public health nurses employed by the Mutsu Municipal Government and staff at workplaces certified for *Sukoyaka* (Health) Support Activity, organised by the Mutsu Municipal Government, informed residents and workers about the Mutsu City Course at presentation meetings and through public relations magazines, local FM radio programmes, flyers posted in local supermarkets and communal facilities, and social networking services. Information in the announcement included the following: ‘Hirosaki University and Mutsu City are going to conduct an intervention study programme that will promote the health of residents in their prime of life.’ In addition, the announcement included preliminary eligibility criteria to screen for potential participants: BMI ≥ 25 kg/m^2^, age 30 to 65 years, and being told to be suffering from a metabolic syndrome by a healthcare provider within the previous 1 year. Those who met these criteria were subjected to the detailed inclusion and exclusion criteria at the briefing stage.

**Figure 1:**
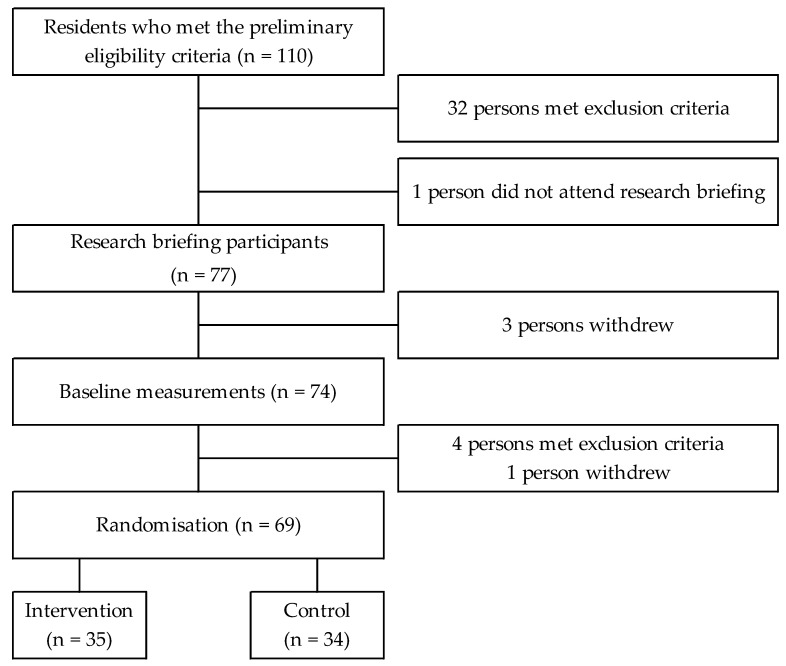
Recruitment flowchart of study participants.

The detailed inclusion criteria were as follows: BMI ≥ 25 kg/m^2^, age 30 to 65 years, did not practise habitual physical activities or sports, had a stable body weight (loss of no greater than 3 kg), took no dietary supplements for weight loss within 1 month before recruitment, were not undergoing pharmacotherapy for diabetes mellitus, non-participation in one or more clinical studies during the past year, and competency to provide informed consent. The principal investigator used the following exclusion criteria to enrol participants: waist circumference >120 cm, pregnant or possibly pregnant, abdominal skin neuropathy, use of any medication that may influence lipid metabolism or body weight, under treatment for and/or history of cerebral and cardiovascular disorders, under treatment for orthopaedic disorders and/or orthopaedic surgery within 1 year, presence of any metal implant, sickness with any abdominal compression, any history of allergy caused by chemical textiles, and any condition that would make participation inappropriate. One hundred and ten residents met the preliminary eligibility criteria as per self-reports. Among them, 77 eligible individuals attended briefing sessions on 6–7 September 2020. Overall, 69 persons agreed to participate in this RCT. All participants provided written informed consent (Figure 1).

### 2.3. Measurement

Anthropometric measurements (height, body weight, waist circumference, and blood pressure) and blood sampling (fasting blood glucose, glycated haemoglobin (HbA1c), triglyceride, and high-density lipoprotein (HDL)-cholesterol) of the intervention and control groups were performed at a community centre for baseline and follow-up data 20 days before the beginning of the intervention and 1 day after the end of the intervention, respectively. Adverse events were identified using a questionnaire during follow-up measurements. A helpline was available at the Department of Health and Beauty Science so the participants could report any adverse events and answer any questions relating to the BCCG.

In addition, eating behaviour and body appreciation were assessed using self-rated questionnaires before and after the intervention. Eating behaviour was assessed using Sakata’s Eating Behaviour Questionnaire, developed to understand the distortions and habits of eating behaviour [14]. It includes 47 (for men) and 45 (for women) items. Each item is rated on a four-point scale ranging from one = ‘there is no such thing’ to four = ‘absolutely’. These questions are classified into seven areas: (1) cognition of constitution, such as ‘I believe that I gain weight because I lie down soon after eating’ [15] and ‘I tend to eat more even if I have a cold’; (2) reasons for eating more, such as ‘I eat my favourite food even after a meal’; (3) eating and drinking because of mood, such as ‘I feel uncomfortable when there are small amounts of food in a refrigerator’; (4) satiety, such as ‘I am not satisfied until I eat to a full stomach’; (5) eating style, such as ‘I stuff my mouth with food when eating’ and ‘little chewing’; (6) meal contents, such as ‘I often eat out or eat delivered food’; and (7) irregular mealtimes, such as ‘I often eat at night’ and ‘I always gain weight after consecutive holidays, the Bon Festival, or the New Year’s holidays.’ Higher scores indicated more inappropriate eating behaviours, and a larger area enclosed by the straight line on a radar chart indicated a larger deviation. Body appreciation was assessed using the body appreciation scale-2 (BAS-2), which has been utilised by researchers to understand the features, correlates, and potential outcomes of a positive body image [16]. Ten items rated on a five-point scale in BAS-2 were scored for the participants. The scale ranged from 1 = never, 2 = seldom, 3 = sometimes, 4 = often, to 5 = always.

### 2.4. Outcomes

The primary outcome was body weight reduction at 12 weeks. The secondary outcomes were improvements in the following items: components of metabolic syndromes (BMI, waist circumference, systolic and diastolic blood pressures, fasting blood glucose, HbA1c, serum triglyceride, and HDL-cholesterol), eating habits as assessed using Sakata’s Eating Behaviour Questionnaire, and body appreciation as assessed using BAS-2. We had initially selected two primary outcomes: body weight reduction and metabolic syndrome. However, we finally chose body weight reduction as the primary outcome and changed the components of metabolic syndrome to secondary outcomes just before randomisation after recruitment owing to a shortage of participants relative to the calculated sample size, due to fear of infection with severe acute respiratory syndrome coronavirus-2 among people in the study area. Therefore, as mentioned below, we utilised metabolic syndrome as a stratification factor for randomisation.

### 2.5. Randomisation

The principal researcher (KI) at the Department of Social Medicine, Hirosaki University, was responsible for randomisation and concealment of the allocation information for randomisation. He assigned participants to the intervention group after randomisation, which was conducted just after the baseline measurement and was performed by a researcher from another department at Hirosaki University. The information of the randomization and group allocation was blinded to the staff hired by the Mutsu Municipal Government who measured body weight and height and gathered related data of the study participants. In our trial with a small number of participants, metabolic syndrome may have affected changes in the obese state and outcome risk. Thus, stratified randomisation was performed, in which all participants in the trial were first grouped into strata of metabolic and non-metabolic syndromes. Subsequently, those in each stratum were grouped into the intervention or control group in a 1:1 ratio using computer-generated random numbers. Owing to the nature of the intervention, participants could not be blinded.

Soon after the randomisation, we mailed all participants to inform them about their allocation group. Participants allocated to the intervention group were also invited to a fitting session. Those allocated to the control group were asked to wait for the invitation to the follow-up measurement session.

### 2.6. Intervention

The intervention comprised BCCG and behaviour change techniques, including self-monitoring, goal setting, and drawing health benefit links with the goals set. The aim of the multi-component intervention was for participants to lose approximately 3% of their body weight in 3 months, which was in accordance with the Guidelines for Management of Obesity Disease issued by the Japan Society for the Study of Obesity [17].

The intervention group was asked to wear the BCCG daily. BCCG is a commercially available clothing (HIGH WAIST BOTTOMS; ATSUGI Co., Ltd., Kanagawa, Japan; $28.60–$30.50 per piece). The clothing is made of a circular-knit elastic fabric of nylon and polyurethane. The upper end of the BCCG reached the hypochondrium, and the main parts of the clothing completely covered the abdomen. The manufacturer notes that the high waist bottom is body-shaped self-monitoring clothes, as it exerts continuous tactile pressure on the abdomen and buttocks. BCCG has four sizes: small, medium, large, and extra-large. BCCGs were individually fitted following the manufacturer’s guidelines (available waist circumference: <120 cm) before the beginning of the intervention. As the mild pressure of the BCCG slightly corrects the hanging lower abdomen and buttocks, wearing the BCCG shapes the body to a certain extent. However, the pressure of the BCCG is minimal compared with that of a tight girdle or corset for body correction and that of elastic stockings for lymphedema. We measured the BCCG pressure on the abdominal skin in a sitting position during the fitting session for each participant using a simple body pressure-measuring device (Palm Q^®^; Cape Corp., Kanagawa, Japan). We set the pressure to <40 hPa, the industrial standard for normal underclothes. Regardless of the actual pressure, a larger BCCG was recommended if the participant’s groin felt strong pressure or was likely to have pain.

They were asked to achieve ten health behaviour goals (four essential goals and six selected goals) in everyday life to reduce their body weight. The essential goals consisted of (1) chewing each bite of food more than 20 times daily, (2) walking faster and taking longer strides, (3) brushing their teeth soon after finishing meals, and (4) looking in a mirror to monitor their complexion, countenance, and body shape. The selected goals consisted of six goals chosen by each participant from a list of 46 predefined goals in three categories (diet, exercise, and sleep/rest) that were expected to reduce body weight. Participants in the intervention group were given a digital weighing scale (HD-661; Tanita Co., Ltd., Tokyo, Japan) to measure body weight daily.

Participants in the intervention group had a 30-min individual session with public health nurses. The nurses explained to the participants the details of our multi-component behaviour change programme, including the expected effects of wearing the BCCG, the essential behavioural goals for behavioural changes, and the instruction to mark the body weight using a sequential line graph on a sheet of paper after measurement. The public health nurses emphasised that a fast weight drop was not preferable, and that goal setting was for behaviour change rather than achieving weight outcomes. Participants in the intervention group were then asked to fill the blanks on a check sheet daily to indicate whether each goal, wearing the BCCG and self-weighing, had been accomplished. The check sheet had an entry column for consultations regarding the BCCG. The column was used to report adverse events if any occurred. Participants were asked to return the check sheets every 2 weeks to the Department of Health and Beauty Science.

At the 6th week, a brief mail survey was conducted to identify persons who wished to wear a smaller or larger BCCG. We sent BCCGs that were one size smaller or larger than the size currently worn by participants according to their requests if possible (e.g., those who had worn the extra-largest size would not have been sent larger BCCGs even if they had requested larger sizes). In the 1st, 5th, and 9th weeks, participants in both groups received a health information booklet (1st week: ‘Review your everyday diet,’ 5th week: ‘Exercise prevents two major bad habits,’ and 9th week: ‘Understand the benefits of reducing salt, and let’s challenge!’. They were encouraged to read booklets on cultivating a mindset to engage in behaviours that promote long-term physical and emotional health. A physician gave the potential participants in the intervention and control groups a lecture on the association between reducing obesity/overweight and health benefits before randomisation.

### 2.7. Statistical Analysis

Data were analysed using STATA 13.1 (StataCorp LLC, College Station, TX, USA). Demographic and clinical characteristics are summarised as counts (percentages) for categorical variables or mean (standard deviation) for normally distributed continuous variables. The primary aim of this study was to determine whether changes in body weight at baseline and the 12th week differed between the intervention and control groups. For the intention to treat, linear mixed models were constructed for analysing the repeated-measure data at baseline and the 12th week. The results of the mixed models demonstrated the interaction terms of time for a group that indicated the effect of time on outcomes. The intervention effect on the primary outcome was quantified using Cohen’s d [18]. The sample size calculation based on a preliminary study indicated that 40 participants per group would improve detection, providing 80% of the power to detect 80% of the intervention group, whereas only 50% of the control group would be detected with a 5% significance level. The final sample size was 50 participants, including the expected dropouts (10 participants) during the 12-week intervention period. The prespecified statistical analyses were conducted by a researcher from another department at Hirosaki University. She moved to the Department of Social Medicine just before the analyses but remained blind to the allocation information until the manuscript was drafted.

For the post-hoc analysis, we constructed a linear mixed model for analysing the 12 repeated-measures data from the 1st week to the 12th week to compare the frequency of wearing the BCCG between participants with 3% or more weight loss and those with 3% or less weight loss in the intervention group.

## 3. Results

In mid-October 2020, 69 participants were randomly assigned to one of two groups: 35 in the intervention group and 34 in the control group. Three of the 35 participants in the intervention group did not complete the measurements in the 12th week because they did not participate in the measurement session. There were no significant differences in the baseline characteristics between the intervention and control groups (Table 1).

Table 2 presents the body weight and other components of metabolic syndrome at baseline and the 12th week among participants with complete data. The change in the body weight of the intervention group from baseline (Table 1, n = 35) to the 12th week was 1.7 kg. The corresponding value in the control group was 0.4 kg. Weight loss, the primary outcome, was different between the intervention and control groups (*p* < 0.05 for the interaction between time and groups in a repeated measures mixed model), giving the effect size of 0.64 (95% confidence interval, 0.50–0.78).

**Figure 2:**
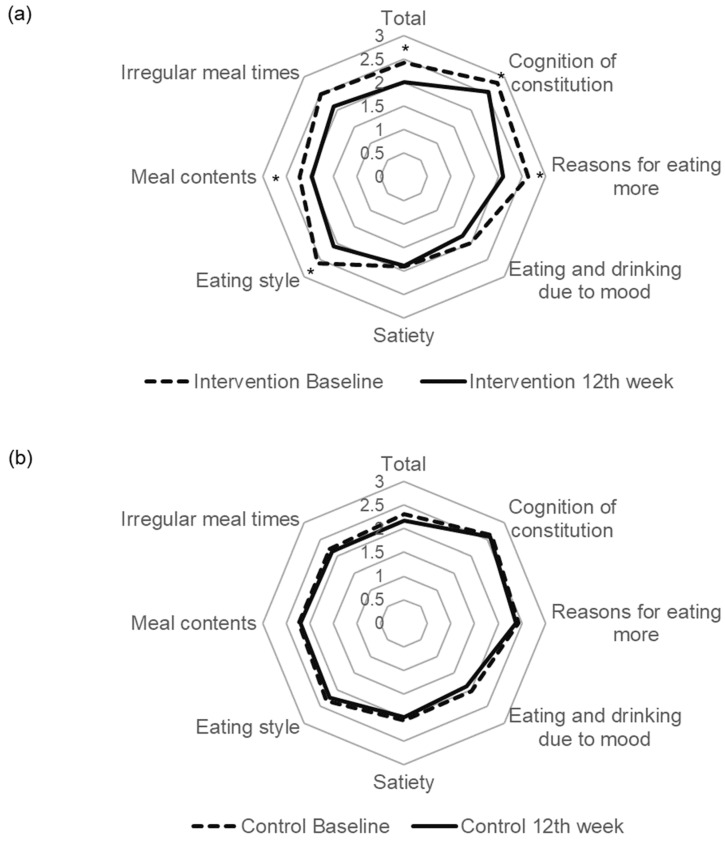
Changes in scores determined using the Sakata’s Eating Behaviour Questionnaire in the intervention and control groups. * *p* < 0.05 for each interaction between time and groups in repeated measures mixed models. (**a**) Intervention group. (**b**) Control group.

A significant difference in BMI changes among secondary outcomes was also observed between the two groups (*p* < 0.05 for the interaction between time and groups in a repeated measures mixed model). Figure 2 shows the mean scores and changes determined using Sakata’s Eating Behaviour Questionnaire in total and in the seven areas at baseline and the 12th week. The total and four subscale scores (cognition of constitution, reasons for eating more, eating style, and meal contents) decreased significantly in the intervention group compared with those in the control group (*p* < 0.05 for each interaction between time and groups in repeated measures mixed models).

The means and changes determined using BAS-2 are summarised in Table 3. At baseline, the BAS-2 total score and scores of the ten items were not significantly different between the groups. However, from baseline to the 12th week, scores of ‘Right now, I feel that my body has at least some good qualities’ and ‘At this moment, I take a positive attitude toward my body’ increased in the intervention group and decreased in the control group (*p* < 0.05, for each interaction term between time and groups in repeated measures mixed models).

**Figure 3:**
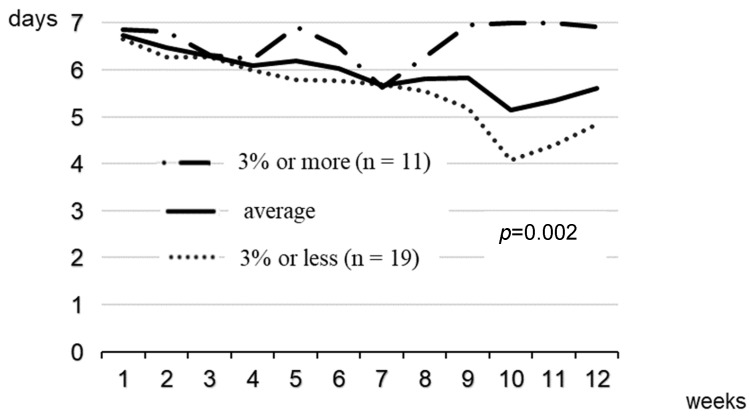
Mean days of wearing BCCG in each week for participants with 3% or more weight loss and those with 3% or less weight loss. BCCG, body compression corrective garment. *p*-value is for the interaction between time and groups in repeated-measures mixed models.

Participants in the intervention group returned most check sheets, which they had been asked to complete, covering, on average, 89.5% of the 84 days during the 12-week intervention period. We performed post-hoc analyses using check sheets. The frequency of wearing the BCCG is shown in Figure 3, where the participants wore the BCCG for almost 7 days in the first week, but the frequency dropped to about 5 days in the 10th week (around the New Year holidays). Among participants who returned the check sheets at the 12th week in the intervention group, 11 with body weight reduction of ≥3% and the other 19 with body weight reduction of ≤3% wore the BCCG an average of 6.9 vs. 4.8 days, respectively. The former wore the BCCG more days than the latter during the 12-weeks intervention period based on the intention-to-treat principle (*p* < 0.05 for the interaction between time and groups in a repeated measures mixed model).

Two participants in the intervention group reported adverse events through the questionnaire at follow-up. Both felt uncomfortable wearing the BCCG, and one experienced redness and itching. However, the latter lost >3% of body weight during the 12-week intervention. The column for consultation on the check sheets had no other adverse events.

## 4. Discussion

In our 12-week RCT using BCCG combined with behaviour change techniques, the reduction in body weight and BMI was greater in the intervention group than in the control group. In addition, the total and four subscale scores of eating behaviour and scores of two items on body appreciation improved significantly in the intervention group compared with those in the control group.

To the best of our knowledge, this study is the first to introduce the use of BCCG in a multi-component behavioural intervention for overweight/obesity, while technology-based modalities such as computer-based modules and various alternative treatments have been previously incorporated into behaviour change programmes [19,20]. Participants in the intervention group lost 1.7 kg of body weight during the 12 weeks of the present study. This corresponds to our primary expectation of a gradual decrease in body weight according to the Japanese Treatment Guidelines for Obesity [17]. The difference in change between the intervention and control groups, 1.3 kg (1.7–0.4 kg), seems greater than the 1.1 kg mean difference obtained by supervised physical activity sessions at 12 months reported in a meta-analysis [21]. The difference in the present study is within the range of 0.3 kg to 4.93 kg, which was obtained after administering dietary supplements and alternative therapies at 12 months [20]. The effect size in the present study, d = 0.64, was medium and significant, greater than that in interventions via mobile phones for weight loss [22]. The intervention combining BCCG and behaviour change techniques in the present study is a new, efficient, and easy-to-maintain approach for weight loss.

It seems easy to continue wearing the BCCG, as the participants wore their clothing for an average of 6 days weekly at the end of the study period. High adherence might have supported the conventional behavioural techniques with a relatively weak intensity adopted in this study, as the intervention group that lost ≥3% of body weight wore BCCG for more days than those who lost ≤3%. The mean difference in body weight loss for the 12 weeks intervention period reached 4.5 kg between those who lost ≥3% and those who lost ≤3% body weight (data not shown). The BCCG, which exerts continuous tactile pressure on the lower body, is likely to give a feeling of fullness earlier during meals to prevent rapid eating. This possibly contributed to the improvement in scores for the eating style subscale of Sakata’s Eating Behaviour Questionnaire as the subscale included questions regarding eating pattern such as ’eat fast’, ‘little chewing’ [14] and ‘stuffing one’s mouth full.’ The tactile pressure of the BCCG was not enough to lead to improvement in the satiety subscale of the Sakata’s questionnaire. It is also possible that the BCCG then gave participants a feeling of success by reducing the tight waist fit that accompanied the reduction in body weight. Even those participants who lost body weight within 3% wore the BCCG for an average of 5 days weekly at the end of the intervention period. Therefore, the influence of continuous tactile pressure on the feeling of fullness cannot completely explain the weight loss caused by this intervention. The intervention as a whole, including wearing BCCG, may change and improve behaviour related to overweight/obesity. However, we should scrutinise the characteristics of those who lost ≥3% body weight to efficiently utilise this multi-component behavioural intervention, including wearing BCCG.

Compared with participants in the control group, those in the intervention group showed improved eating behaviour, assessed using Sakata’s Eating Behaviour Questionnaire. The scores for the three subscales: ‘cognition of constitution’, ‘reasons for eating more’, and ‘meal contents’ in addition to ‘eating style’, of the questionnaire were higher in the intervention group. The subscales are associated with perception, attitude, and practice relating to obesity. Eating pattern change caused by the BCCG occurred first, as mentioned above. Noticing gradual weight loss through self-monitoring with weighing and marking the body weight using sequential lines, might improve distorted perceptions of constitution being likely to be obese, as the participants realised the relationship between wearing BCCG days and weight loss. Practising wearing the BCCG and improved perception might contribute to shifting attitudes toward overeating and unhealthy eating practices such as choosing junk food and fast food. Weight loss in the intervention group may be partly attributed to the improvement of eating behaviours, while the intervention included goal setting for behavioural changes in physical activities and sleeping/resting. The present study was conducted from the second semester in year 2020 to the beginning of 2021, in the middle of the coronavirus disease (COVID-19) pandemic. Although the severe acute respiratory syndrome coronavirus 2 (SARS-CoV-2) infection rates in 2020 fiscal year were low and mandatory lockdowns had not been imposed in Japan, people reduced outdoor activities following the campaign of ‘avoid three Cs (crowded places, close-contact setting, and confined and enclosed spaces)’ [23,24]. Therefore, it was highly unlikely that the participants in both groups had increased physical activity and reduced sedentary time. As people in Japan also likely decreased eating-out to prevent infection with SARS-CoV-2, the equal probability of decreasing eating-out should have been expected in the two groups of the present study under randomisation. Nevertheless, the scores for the meal contents subscale, which involve ‘eating-out’ and ‘fast food’, decreased more in the intervention group than the control group.

To some extent, participants in the intervention group improved their body image, assessed using BAS-2. Participants might realise their weight loss through weigh-in, a part of self-monitoring performed by looking at a mirror, an essential goal set at the beginning of the study. They might realise the correction of their hanging lower abdomen and buttocks in the BCCG with mild pressure and consequently the change in their body shape. Weight loss and/or awareness of changes in body shape might have improved the participants’ body image. Improvement in BAS-2 scores may reflect a conscious feeling for body shape. Consciousness may facilitate sustainable efforts to change lifestyles. Higher levels of body appreciation were reported to be linked to higher intuitive eating and psychological well-being, such as self-esteem and proactive coping [16]. Given the undeniable differences in social perspectives, expectations, and pressures regarding body composition and body image according to sex, women participants could have looked in the mirror more than men. However, we found no sex difference in the frequency of looking in a mirror in the intervention group (data are not shown in the Results section because this was outside the scope of the present study). Although we could not find a statistically significant difference between sexes, the proportions of participants with body weight reduction of ≥3% in the intervention group were 33.3% in men and 23.5% in women (data also not shown). We suppose that most male participants wore a body compression garment for the first time and that the new experience motivated them to continue wearing the BCCG and, accordingly, were more likely to succeed in reducing their weight. Notably, the health promotion sectors often fail to involve men in these studies, while our study had an equal number of men and women. Our weight reduction programme, including the BCCG, seemed to effectively motivate men and women to begin reducing weight.

The present study has some limitations. First, the evaluation of changes in body weight in a 3-month study may be insufficient. We believe that more weight loss would have occurred if the intervention period was longer. Second, we did not examine whether weight loss was sustained for at least 12 months [19]. Traditional weight reduction sessions still cannot overcome the major weakness of existing post-treatment weight regain [8]. Feeling the body at all times of the day by wearing the BCCG would lead to finding, noticing, and having a chance to change attitudes to advance health in the near future. We expect that the effect of BCCG would maintain a longer weight loss than other behavioural change programmes. Third, we could not follow up with three participants in the intervention group. As the intervention group had only 35 participants, the dropout of the three participants could affect the results. However, we statistically adjusted for the effect of dropout using repeated measure mixed models. We made enormous efforts to follow up; however, the three individuals could not participate in outcome measurement because of self-restriction to prevent SARS-CoV-2 infection as Japan’s government declared the COVID-19-related state of emergency for Tokyo and neighbouring prefectures 700 km away from the study area just 10 days before the outcome measurement session. There were no other reasons, such as adverse events or ineffectiveness of the intervention programme for weight loss, to prohibit these individuals from participating in the outcome measurement. We do not know the reasons dropouts occurred only in the intervention group. Randomisation might not be able to control for unknown confounders in a small sample study. The findings of the present study should be confirmed in a future RCT with a longer intervention period, longer follow-up period, and larger sample size.

## 5. Conclusions

We showed that our newly developed 3-month intervention combining the BCCG with a behaviour change programme was effective for overweight/obese individuals. Wearing BCCG more often increased weight loss. The intervention programme improved eating behaviour, especially eating patterns, distorted perception, attitude toward overeating, and unhealthy eating practices relating to obesity. In combination with low-intensity conventional behavioural techniques, the BCCG, which was developed to integrate positive appearance, good health, and cognitive–behaviour effects, may contribute to changes in eating behaviour and subsequently cause weight loss.

## Figures and Tables

**Table 1:** Characteristics of study participants at baseline.

	Intervention (n = 35)		Control (n = 34)	
Sex	n (%)		n (%)	
Men	18 (51.4)		17 (50.0)	
Women	17 (48.6)		17 (50.0)	
	Mean	SD	Mean	SD
Age	45.3	8.2	44.9	9.1
Height (cm)	165.4	8.6	165.2	9.8
Weight (kg)	79.2	11.7	80.2	13.9
BMI (kg/m^2^)	30.1	7.7	29.2	2.6
Waist circumference (cm)	99.1	6.3	98.9	7.7
Glucose (mg/dL)	93.9	26.5	95.3	16.3
HbA1c (%)	5.6	1.0	5.7	0.7
SBP (mmHg)	139.3	17.0	134.4	20.4
DBP (mmHg)	91.1	11.1	85.7	15.3
Triglyceride (mg/dL)	188.8	127.7	165.5	98.0
HDL-cholesterol (mg/dL)	52.5	13.7	53.1	12.4

BMI, body mass index; DBP, diastolic blood pressure; HbA1c, glycated haemoglobin; HDL, high-density lipoprotein; SBP, systolic blood pressure; SD, standard deviation.

**Table 2:** Body weight and other components of metabolic syndrome at baseline and the 12th week among participants with complete data.

	Intervention (n = 32) ^†^		Control (n = 34)	
	Mean	SD	Mean	SD
Body weight (kg)				
Baseline	79.2	11.8	80.2	13.7
12th week	77.5 ^‡^	12.0	79.8	13.8
BMI (kg/m^2^)				
Baseline	28.9	2.4	29.2	2.6
12th week	28.3 ^‡^	2.5	29.1	2.6
Waist circumference (cm)				
Baseline	99.2	6.4	98.9	7.6
12th week	95.9	7.5	97.4	8.6
Systolic blood pressure (mmHg)				
Baseline	139.4	17.5	134.4	20.1
12th week	137.8	15.5	135.3	20.0
Diastolic blood pressure (mmHg)				
Baseline	90.8	11.3	85.7	15.1
12th week	90.1	12.0	86.6	14.3
Glucose (mg/dL)				
Baseline	93.1	26.5	94.2	15.0
12th week	94.2	30.5	98.3	34.7
HbA1c (%)				
Baseline	5.6	1.1	5.7	0.6
12th week	5.5	1.0	5.8	0.8
Triglyceride (mg/dL)				
Baseline	181.0	123.6	165.5	98.0
12th week	146.3	94.4	150.7	110.1
HDL-cholesterol (mg/dL)				
Baseline	53.6	13.5	53.1	12.4
12th week	54.8	12.1	53.8	13.7

^†^, the number of participants in the intervention group was 35 at baseline and 32 at the 12th week. ^‡^, *p* < 0.05 for differences in changes in measures between intervention (n = 35) and control (n = 34) groups using a repeated measures mixed model. BMI, body mass index; HbA1c, glycated haemoglobin; HDL, high-density lipoprotein; SD, standard deviation.

**Table 3:** Scores on the Body Appreciation Scale-2 at baseline and the 12th week.

	Baseline				12th Week			
	Control	SD	Intervention	SD	Control	SD	Intervention	SD
BAS-2 total score	23.3	8.1	22.7	7.3	−1.0	5.1	1.2	7.4
1. Right now, I respect my body.	1.5	0.8	1.4	0.8	0.2	0.5	0.2	0.84
2. At this moment, I feel good about my body.	2.1	0.8	2.1	1.1	0.0	0.8	0.0	1.3
3. Right now, I feel that my body has at least some good qualities. *	2.7	1.3	2.6	1.1	−0.1	0.9	0.2	1.3
4. At this moment, I take a positive attitude toward my body. *	2.5	1.2	2.7	1.1	0.0	1.1	0.5	1.3
5. Right now, I am attentive to my body’s needs.	2.7	1.2	2.6	1.1	−0.2	1.1	0.0	1.3
6. At this moment, I feel love for my body.	2.7	1.2	2.7	1.0	0.0	1.0	0.2	1.2
7. Right now, I appreciate the different and unique characteristics of my body.	2.3	1.2	2.2	1.1	0.3	1.1	0.3	0.9
8. At this moment, my behaviour reveals my positive attitude toward my body; for example, I hold my head high and smile.	2.4	1.1	2.2	1.1	−0.2	0.9	0.3	1.2
9. Right now, I am comfortable in my body.	2.1	0.9	1.9	1.0	−0.2	0.9	−0.1	0.9
10. At this moment, I feel like I am beautiful even if I am different from media images of attractive people (e.g., models and actresses/actors).	2.4	1.2	2.5	1.1	−0.2	0.9	−0.2	1.3

* *p* < 0.05, for each interaction between time and groups in repeated measures mixed models. BAS-2, body appreciation scale-2; SD, standard deviation

## Data Availability

The data presented in this study are available upon request from the Department of Social Medicine, Graduate School of Medicine, Hirosaki University, Aomori, Japan (contact via e-mail: soc-med@hirosaki-u.ac.jp) for researchers who meet the criteria for access to the data. Researchers must be approved by the research ethics review boards of their affiliation organisations. The data cannot be shared publicly because of ethical concerns.
