# Correction: Kanda et al. Body Compression Corrective Garment and Eating Behavioural Change for Weight Reduction: The Mutsu City Randomised Controlled Trial. *Healthcare* 2023, *11*, 942

**DOI:** 10.3390/healthcare12020210

**Published:** 2024-01-15

**Authors:** Akira Kanda, Yoshikuni Sugimura, Hideki Ohishi, Satoru Tatebayashi, Kaori Sawada, Kyi Mar Wai, Kei Nishiguchi, Asano Tanabu, Songee Jung, Koichi Murashita, Shigeyuki Nakaji, Kazushige Ihara

**Affiliations:** 1 Department of Health and Beauty Science, Graduate School of Medicine, Hirosaki University, Aomori 036-8562, Japan; a_kanda@auhw.ac.jp (A.K.); h.ooishi@atsugi.co.jp (H.O.); nakaji@hirosaki-u.ac.jp (S.N.); 2 Department of Nutrition, Faculty of Health Sciences, Graduate School of Health Sciences, Aomori University of Health and Welfare, Aomori 030-8505, Japan; 3 Department of Social Medicine, Graduate School of Medicine, Hirosaki University, Aomori 036-8562, Japan; y.sugimura@hirosaki-u.ac.jp (Y.S.); iwane@hirosaki-u.ac.jp (K.S.); kyimar@hirosaki-u.ac.jp (K.M.W.); h17gm141@hirosaki-u.ac.jp (A.T.); 4 Department of Innovation Center for Health Promotion, Graduate School of Medicine, Hirosaki University, Aomori 036-8562, Japan; 5 Atsugi Corporation, Kanagawa 243-0493, Japan; s.tatebayashi@atsugi.co.jp; 6 Atsugi Tohoku Mutsu Office, Aomori 035-0061, Japan; kei.nishiguchi.3j@hirose-gl.com; 7 Department of Digital Nutrition and Health Sciences, Graduate School of Medicine, Hirosaki University, Aomori 036-8562, Japan; jonsoni@hirosaki-u.ac.jp; 8 COI Research Initiatives Organization, Graduate School of Medicine, Hirosaki University, Aomori 036-8562, Japan; murasita@hirosaki-u.ac.jp

## Error in Figure

In the original publication [1], there was a mistake in Figure 3 as published. The figure legends for the ‘average’ group and the ‘less than 3%’ group were reversed. The line for the ‘average’ group should appear between the ‘more than 3%’ group and the ‘less than 3%’ group. The corrected Figure 3 appears below.

The authors state that the scientific conclusions are unaffected. This correction was approved by the Academic Editor. The original publication has also been updated.

## Figures and Tables

**Figure 3:**
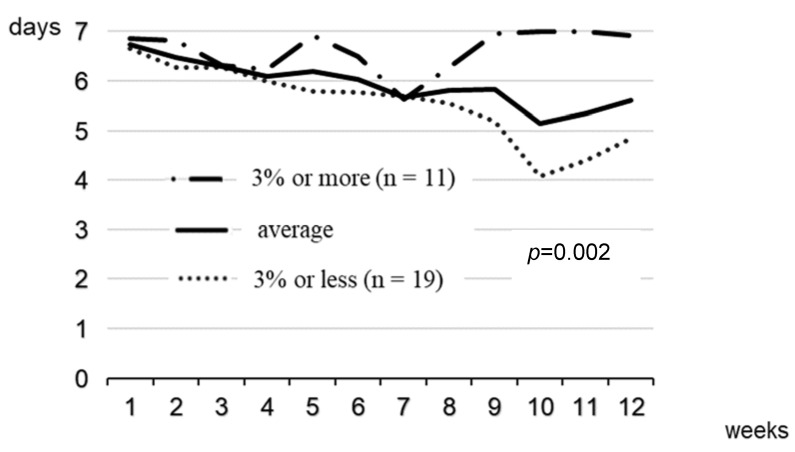
Mean days of wearing BCCG in each week for participants with 3% or more weight loss and those with 3% or less weight loss. BCCG, body compression corrective garment. *p*-value is for the interaction between time and groups in repeated-measures mixed models.

## References

[1] Kanda A., Sugimura Y., Ohishi H., Tatebayashi S., Sawada K., Wai K.M., Nishiguchi K., Tanabu A., Jung S., Murashita K. (2023). Body Compression Corrective Garment and Eating Behavioural Change for Weight Reduction: The Mutsu City Randomised Controlled Trial. Healthcare.

